# Increased threat of urban arboviral diseases from *Aedes aegypti* mosquitoes in Colombia

**DOI:** 10.1016/j.ijregi.2024.100360

**Published:** 2024-03-22

**Authors:** Rosa Margarita Gélvez Ramírez, Chloé Bohers, Laurence Mousson, Yoann Madec, Marie Vazeille, Géraldine Piorkowski, Sara Moutailler, Francisco J Diaz, Guillermo Rúa-Uribe, Luis Angel Villar, Xavier de Lamballerie, Anna-Bella Failloux

**Affiliations:** 1 Unité des Virus Émergents (UVE: Aix-Marseille Univ-IRD 190-Inserm 1207), Marseille, France; 2 Centro de Atención y Diagnóstico de Enfermedades Infecciosas-CDI, Grupo INFOVIDA, Bucaramanga, Colombia; 3 Institut Pasteur, Université de Paris, Unit of Arboviruses and Insect Vectors, Paris, France; 4 Institut Pasteur, Université de Paris, Epidemiology of Emerging Diseases unit, Paris, France; 5 UMR BIPAR, Animal Health Laboratory, ANSES, INRA, Ecole Nationale Vétérinaire d'Alfort, Université Paris-Est, Maisons-Alfort, France; 6 Grupo de Inmunovirología, Facultad de Medicina, Universidad de Antioquia, Medellín, Colombia; 7 Grupo Entomología Médica, Facultad de Medicina, Universidad de Antioquia, Medellín, Colombia

**Keywords:** Aarbovirus, *Aedes aegypti*, Colombia, Vector competence

## Abstract

**Objectives:**

Our study targets the potential of the local urban mosquito *Aedes aegypti* to experimentally transmit chikungunya virus (CHIKV), dengue virus (DENV), yellow fever virus (YFV), and Zika virus (ZIKV).

**Methods:**

We collected eggs and adults of *Ae. aegypti* in Medellín, Colombia (from February to March 2020) for mosquito experimental infections with DENV, CHIKV, YFV and ZIKV and viral detection using the BioMark Dynamic arrays system.

**Results:**

We show that *Ae. aegypti* from Medellín was more prone to become infected, to disseminate and transmit CHIKV and ZIKV than DENV and YFV.

**Conclusions:**

Thus, in Colombia, chikungunya is the most serious threat to public health based on our vector competence data.

## Highlights

• Chikungunya virus (CHIKV) and Zika virus (ZIKV) better infect *Aedes aegypti*.
• CHIKV and ZIKV better disseminate in *Ae. aegypti*.
• CHIKV and ZIKV are well transmitted by *Ae. aegypti* compared to dengue virus and yellow fever virus.
• Viral particles in abdomen re not an indicator of dissemination and transmission.

## Introduction

Vector-borne disease transmission depends on complex interactions between the vector, the agent, the vertebrate host, and the environment [1]. Arboviruses are naturally cycling between arthropod vectors and susceptible vertebrate hosts. A mosquito acquires the status of vector when it can transmit the virus to a suitable host after ingestion of the virus from an infected host. In the mosquito, the ingested virus infects and replicates in mosquito midgut epithelial cells and after several days of incubation, newly produced virions are released in the mosquito general cavity. With the hemolymph, viral particles disseminate and infect several internal organs and tissues including the salivary glands where the virus replicates and is excreted with saliva during blood feeding [2].

Chikungunya (CHIKV), dengue (DENV), yellow fever (YFV) and Zika (ZIKV) are four arboviruses transmitted by the anthropophilic mosquito *Aedes aegypti* [3]. Originating from rainforests in Africa, *Ae. aegypti* became highly domesticated; this mosquito is a commensal species of humans, adapted to breed in artificial containers, getting blood exclusively on humans, and characterized by a limited flight range. *Ae. aegypti* proliferates in human settings where it acts as the main vector of several arboviruses. Its geographical distribution covers the tropical belt because of the eggs’ resistance to desiccation which fosters long-distance transportation [4].

South America has experienced recent incursions of DENV, CHIKV, and ZIKV in addition to the historical YFV which mainly today circulates in the wild. YFV was brought from Africa to the New World along with its vector *Ae. aegypti* via the slave trade [5], causing urban epidemics. Yellow fever outbreaks were successfully controlled after the discovery of *Ae. aegypti* role in YFV transmission by Finlay and Reed [6]. Longtime restricted to Asia, DENV was suspected in the French West Indies and Panama in the 17^th^ century. DENV is now present in almost all countries in the tropical America with an estimated 2.4 million cases in 2015. In 2013, CHIKV was detected for the first time on Saint Martin Island, and by the end of 2015, it was reported in 50 countries and territories in the Caribbean, and Continental America. In 2013-2014, ZIKV arrived in Brazil and spread to Latin America and the Caribbean. In South America, ZIKV was newly associated with severe manifestations, Guillain-Barré syndrome and microcephaly in newborns [7].

Colombia with its 50 million inhabitants is one of the most affected countries in the American region for arboviruses. *Ae. aegypti* intervenes in arbovirus urban cycle, multiple other mosquito species may have potential to transmit arboviruses and to maintain sylvatic cycles. *Ae. aegypti* is well established in the city of Medellín which experiences high incidences of DENV cases, mainly in the Northeastern part of the city [8]. Our study examines the potential of the local urban mosquito *Ae. aegypti* to transmit experimentally CHIKV, DENV, YFV and ZIKV, and opens discussions on the risk of endemization of these four arboviruses in Colombia.

## Materials and methods

### Mosquito collections and rearing

In the Northeastern part of Medellín, five communes were selected for mosquito collections: Popular, Santa Cruz, Manrique, Aranjuez, and La Candelaria (Supplementary Table 1). From February to March 2020 wild eggs were collected using ovitraps and adults were captured using the Prokopack aspirator. On Institut Pasteur obtained larvae and was constituted the Northeastern population. Generation F3 mosquitoes were used for experimental infections.

### Viral strains

CHIKV 06.21 ECSA lineage (AM258992) (E1-A226V) isolated in 2005 on La Réunion. DENV-1 (MH279620) was isolated in 2009 in French Guiana. YFV-Bolivia American II genotype (MF004382) was isolated in 1999 in Bolivia. ZIKV Martinique Asian genotype (KU647676) was isolated in 2015 in Martinique. These viral strains differed in sequences with strains circulating in Colombia: between 0.2% (ZIKV) and 10.4% (YFV) in nucleotide sequences, and between 0.0% (ZIKV) and 1.08% (CHIKV) in amino acid sequences of envelope proteins. For YFV, there are no recent sequences available for comparison (Supplementary Table 2).

### Mosquito experimental infections

Batches of 1-week-old females were fed through a pig intestine membrane (Hemotek Ltd, Blackburn, UK). The titer of the bloodmeal was 10^7^ FFU/mL for all four viruses. Engorged females were transferred in cardboard containers until examination.

### Analysis of vector competence

Females were analyzed at 7, 14, and 21 days post-infection (dpi). Infection rate (IR) corresponds to the proportion of mosquitoes with an infected abdomen among all engorged mosquitoes. Dissemination rate (DR) refers to the proportion of mosquitoes with infected head and thorax (HT) among mosquitoes with infected midgut. Transmission rate (TR) is the proportion of mosquitoes with infectious saliva among mosquitoes with infected HT [9].

### Viral titration

For mosquitoes exposed to CHIKV, DENV and YFV were titrated by focus fluorescent assay on C6/36 cells. After serial dilutions, samples were inoculated onto C6/36 cells in 96-well plates. After a 3-day incubation period for CHIKV, 5-day for DENV and YFV and 7-day for ZIKV at 28°C, cells were stained with a primary antibody specific to each virus (provided by the French National Reference Center for Arbovirus at the Institut Pasteur for CHIKV, Ms DENV complex MAB 8705 (Millipore, MA, USA) for DENV, and specific primary antibody (catalog number: NB100-64510, Novusbio, CO, USA) for YFV). The secondary antibody was Alexa Fluor 488 goat anti-mouse immunoglobulin G (Life Technologies, CA, USA). For mosquitoes exposed to ZIKV, homogenates were serially diluted and inoculated onto monolayers of Vero cells in 96-well plates. After a 7-day incubation period at 37°C, cells were stained with a solution of safranine (0.5% in 10% formaldehyde and 10% ethanol). Saliva was titrated on monolayers of Vero cells in 6-well plates incubated 7 days under an agarose overlay. Presence of viral particles was assessed by CPE detection.

### Detection of arboviruses in field-collected mosquitoes

After collection on the field, pools of abdomen were constituted, and ribonucleic acid (RNA) was extracted with the Nucleospin RNA extraction kit (Macherey-Nagel, Hoerdt, France). RNA was retro-transcribed using the qScript cDNA Supermix kit (Quanta Biosciences, Beverly, USA). Obtained cDNAs were pre-amplified with the Perfecta Preamp Supermix (Quanta Biosciences, MA, USA) kit [10]. cDNAs were tested in the chip based on the BioMark Dynamic arrays system (Fluidigm Corporation) which targets 95 different genotypes/serotypes of 37 viral species [10].

### Statistical analysis

Rates (infection, dissemination and transmission) were described using median and inter-quartile range. The effect of virus, dpi and viral titer on rates was investigated using logistic regression models. All factors that showed a significant effect in univariate analysis were considered in multivariate analysis to estimate the effect of each factor after adjusting for the other factors considered. Two-by-two interaction between population, virus and dpi was systematically investigated. Viral titer, after being log-transformed to fit the model requirements, was compared between groups using linear regression models. Receiver operating characteristic (ROC) curves were used to evaluate the ability of viral titer to discriminate between mosquitoes showing dissemination on the one hand and transmission capacity on the other hand from the others and identify the best threshold for this. Statistical analyses were conducted using the Stata software (StataCorp LP, Texas, USA) and *P*-values <0.05 were considered significant.

## Results

### CHIKV and ZIKV better infect Ae. aegypti

**Figure 1:**
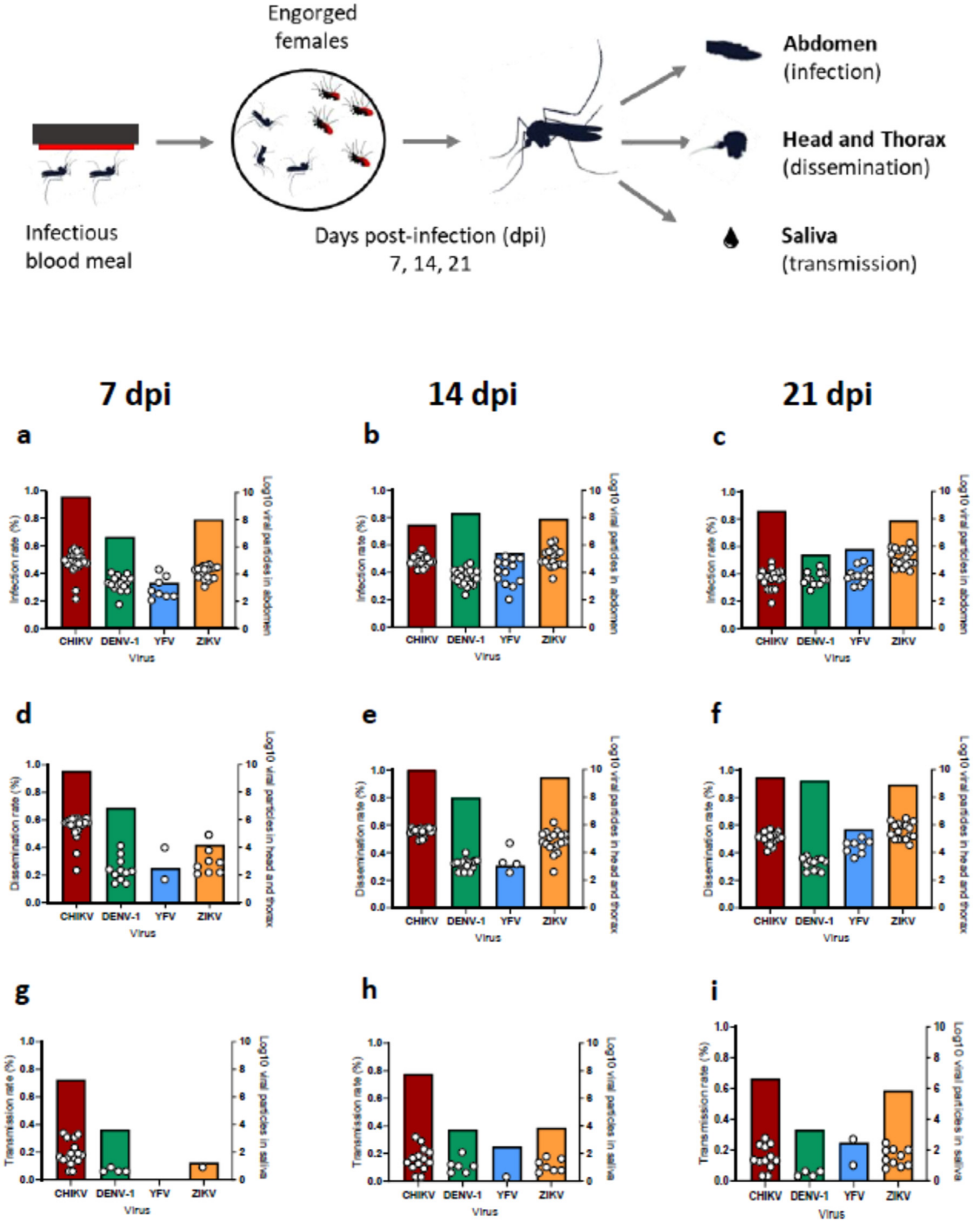
Infection (a-c), dissemination (d-f) and transmission (g-i) rates of *Aedes aegypti* Northeastern exposed to blood meals containing CHIKV, DENV-1, YFV and ZIKV at a titer of 10^7^ FFU/mL. Mosquitoes were examined at 7, 14, and 21 days post-infection. Abdomen, head and thorax (HT), and saliva were titrated to estimate infection, dissemination, and transmission, respectively. IR refers to the proportion of mosquitoes with infected abdomen (i.e. infected midgut). DR corresponds to the proportion of mosquitoes with viral particles detected in HT among mosquitoes with infected abdomen. TR gives the proportion of mosquitoes with viral particles in saliva among mosquitoes with infected HT. Bars corresponds to rates (%) and dots to number of viral particles (Log_10_).

CHIKV, chikungunya virus; DENV, dengue virus; DR, dissemination rate; FFU, focus-forming unit; HT, head and thorax; IR, infection rate; TR, transmission rate; YFV, yellow fever virus; ZIKV, Zika virus.

A total of 201 mosquitoes (70.3%) were infected (Figure 1a-c). IR varied according to the virus (*P <* 0.0001) but not according to the dpi (*P =* 0.78) (Table 1). IR was significantly higher when mosquitoes were infected with CHIKV (85.7%), ZIKV (79.2%) and DENV (68.1%) as compared to YFV (48.6%) (Table 1). Analyses stratified by virus showed that the effect of dpi was always not significant.

**Table 1:** Identification of factors associated with infection rate (IR) (univariate logistic regression models).

	N	IR (%)	Crude OR (95% CI)	*P*
Virus				**< 0.001**
CHIKV	70	60 (85.7)	**6.34 (2.81-14.31)**	
DENV	72	49 (68.1)	**2.25 (1.14-4.34)**	
YFV	72	35 (48.6)	1^a^	
ZIKV	72	57 (79.2)	**4.01 (1.93-8.36)**	
Dpi				0.78
7	96	66 (68.7)	1^a^	
14	96	70 (72.9)	1.22 (0.66-2.28)	
21	94	65 (69.1)	1.02 (0.55-1.88)	

CI, confidence interval; Dpi, day post-infection; DR, dissemination rate; N, number of mosquitoes tested; OR, odds ratio.

a Reference; in bold: significant *P*-values.

When analyzing the viral titer in abdomen, in the 201 mosquitoes with infection, using a linear regression model, abdomen titer increased with dpi for YFV (*P =* 0.001), and ZIKV (*P <* 0.001) while it remained stable whatever dpi for DENV (*P =* 0.29) and it decreased for CHIKV (*P <* 0.001) (Supplementary Table 3). At 21 dpi, abdomen titer was significantly different between viruses (*P <* 0.001), however, two-by-two comparisons showed that only ZIKV had a significantly higher abdomen titer (*P <* 0.001), all other comparisons being non-significant (p > 0.05) (Supplementary Table 4).

### CHIKV and ZIKV better disseminate in Ae. aegypti

Among the 201 mosquitoes infected, viral dissemination was detected in 154 (76.6%) (Figure 1d-f). To investigate whether abdomen titer could discriminate mosquitoes with dissemination from those without, a ROC curve was built (Supplementary Figure 1a). The area under the curve (AUC) value was equal to 0.70 showing that no threshold efficiently discriminated between mosquitoes with and without dissemination. As a consequence, abdomen titer was dichotomized based on the median value, but as abdomen titer distribution was different according to the virus, dichotomization was performed using virus-specific medians. In univariate analysis, DR significantly differed according to virus (*P <* 0.001), dpi (*P =* 0.024) and abdomen titer (*P <* 0.001) (Table 2).

**Table 2:** Identification of factors associated with DR (univariate and multivariate logistic regression models).

	N	DR (%)	Crude OR (95% CI)	p	Adjusted OR (95% CI)	*P*
**Virus**				**< 0.001**		**< 0.001**
CHIKV	60	58 (96.7)	**43.50 (9.11-207.71)**		**74.91 (14.46-387.95)**	
DENV	49	39 (79.6)	**5.85 (2.22-15.42)**		**10.00 (3.28-30.47)**	
YFV	35	14 (40.0)	1^a^		1^a^	
ZIKV	57	43 (75.4)	**4.61 (1.86-11.40)**		**7.96 (2.72-23.32)**	
**Dpi**				**0.024**		**0.021**
7	66	43 (65.1)	1^a^		1^a^	
14	70	56 (80.0)	2.14 (0.99-4.64)		**2.82 (1.06-7.52)**	
21	65	55 (84.6)	**2.94 (1.27 -6.83)**		**4.34 (1.46-12.93)**	
**Abdomen titer**^b^				**0.003**		**0.012**
<median	94	63 (67.0)	1^a^		1^a^	
≥ median	107	91 (85.0)	2.80 (1.41-5.54)		**3.03 (1.28-7.19)**	

CI, confidence interval; Dpi, day post-infection; DR, dissemination rate; N, number of mosquitoes tested; OR, odds ratio.

a Reference; in bold: significant *P*-values.

b Dichotomization based on the virus-specific median values.

In multivariate analysis, virus (*P <* 0.001), dpi (*P =* 0.021) and abdomen titer (*P =* 0.012) all remained independently associated with DR (Table 2). As compared to YFV, DR was significantly larger with CHIKV, DENV and ZIKV. DR was also larger at 14 and 21 dpi as compared to 7 dpi. DR was also larger in mosquitoes with abdomen titer above the median as compared to those with an abdomen titer below the median. To illustrate these results, at 21 dpi, DR was 94.73% (N = 19) for CHIKV, 92.30% (N = 13) for DENV, 89.47% (N = 19) for ZIKV, and 57.14% (N = 14) for YFV (Supplementary Table 5).

When analyzing the viral titer in HT titer, in the 154 mosquitoes with dissemination, using a linear regression model, it significantly increased with dpi for DENV (*P <* 0.009), YFV (*P =* 0.002), and ZIKV (*P <* 0.001) while it remained steady whatever dpi for CHIKV (*P =* 0.69) (Supplementary Table 4). Overall, HT titer differed between viruses (*P <* 0.001). At 21 dpi, HT titer was significantly different between the four viruses (*P <* 0.001); two-by-two comparisons showed that HT titer was significantly lower with DENV as compared with YFV, CHIKV and ZIKV (all three *P <* 0.001), and that HT titer was significantly lower with YFV as compared with ZIKV (*P =* 0.002) (Supplementary Table 4).

### CHIKV and ZIKV are well transmitted by Ae. aegypti compared to DENV and YFV

Among the 154 mosquitoes presenting disseminated infection, 77 (50.0%) were able to transmit the virus (Figure 1g-i). To investigate whether HT titer could discriminate the mosquitoes able to transmit from those not able to, ROC curves were built (Supplementary Figure 1b). The AUC value was 0.74 showing that no threshold efficiently discriminated between mosquitoes able and not able to transmit. As a consequence, HT titer was dichotomized simply based on the virus-specific medians. In univariate analysis, TR significantly differed according to the virus (*P =* 0.0001), abdomen titer (*P =* 0.009), HT titer (*P <* 0.0001) but not according to the dpi (*P =* 0.98) (Table 3).

**Table 3:** Identification of factors associated with TR (univariate and multivariate logistic regressions).

	N	TR (%)	Crude OR (95% CI)	p	Adjusted OR (95% CI)	p
**Virus**				**< 0.001**		**< 0.001**
CHIKV	58	42 (72.4)	**9.63 (2.37-39.05)**		**14.91 (3.36-66.32)**	
DENV	39	14 (35.9)	2.05 (0.49-8.62)		2.68 (0.60-11.91)	
YFV	14	3 (21.4)	1^a^		1^a^	
ZIKV	43	18 (41.9)	2.64 (0.64-10.85)		3.10 (0.72-13.40)	
**Dpi**				0.98		
7	43	21 (48.8)	1^a^			
14	56	28 (50.0)	1.05 (0.47-2.32)			
21	55	28 (50.9)	1.08 (0.49-2.41)			
Abdomen titer^b^				**0.033**		0.06
<median	63	25 (39.7)	1^a^		1^a^	
≥median	91	52 (57.1)	**2.03 (1.05-3.90)**		2.23 (0.96-5.23)	
HT titer b				**0.006**		0.08
<median	73	28 (38.4)	1^a^		1^a^	
≥median	81	49 (60.5)	**2.46 (1.29-4.71)**		2.03 (0.91-4.54)	

CI, confidence interval; Dpi, day post-infection; DR, dissemination rate; N, number of mosquitoes tested; OR, odds ratio.

a Reference; in bold: significant *P*-values.

b Dichotomized based on virus-specific median values.

In multivariate analysis, TR was significantly different between virus (*P <* 0.001) (Table 3). TR was significantly higher with CHIKV compared to the other three viruses. After adjusting for virus, HT titer and abdomen titer only tended to be associated with TR (*P =* 0.06 and *P =* 0.08, respectively). The odds of transmission tended to be higher in mosquitoes with a titer above he median. TR was significantly higher with CHIKV compared to the other three viruses; as an example, at 21 dpi, TR was equal to 66.66% (N = 18) for CHIKV, 58.82% (N = 17) for ZIKV, 33.33% (N = 12) for DENV and 25% (N = 8) for YFV (Supplementary Table 5).

Because the number of mosquitoes able to transmit was small (n = 77) and the residuals after linear regression did not follow a Gaussian distribution, the use of non-parametric tests was more appropriate to evaluate the effect of the covariates on the number of viral particles in saliva (saliva titer). After excluding the three mosquitoes with YFV-infected saliva, saliva titer significantly differed by virus (*P <* 0.001). On the other hand, saliva titer was not influenced by dpi (*P =* 0.60) nor by abdomen or HT titers when dichotomized (*P =* 0.93 and *P =* 0.38, respectively). The main difference was then observed between mosquitoes infected with DENV that presented lower saliva titer than mosquitoes infected with CHIKV and ZIKV.

Collectively, it appears that after dissemination, CHIKV and ZIKV were more efficient to be transmitted by *Ae. aegypti* Northeastern with higher TRs and saliva titers.

### The number of viral particles in abdomen is not a good indicator of successful viral dissemination and transmission

**Figure 2:**
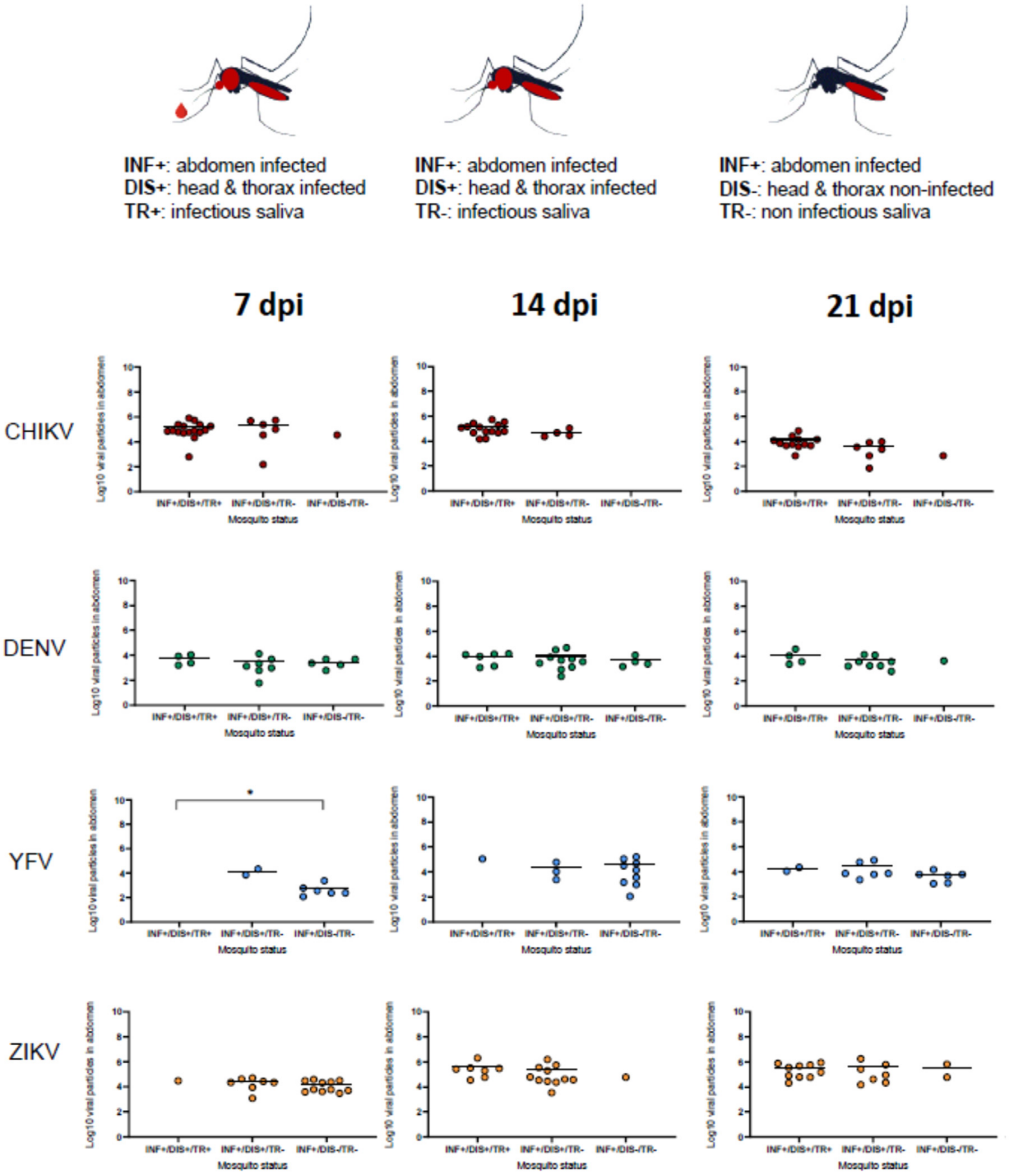
Viral loads in mosquito abdomen according to days post-infection (dpi), virus (CHIKV, DENV, YFV, and ZIKV), and mosquito status (INF+/DIS+/TR+, INF+/DIS+/TR-, INF+/DIS-/TR-). INF+ refers to mosquitoes with an infected midgut. DIS+/DIS- corresponds to mosquitoes with head and thorax infected or non-infected, respectively. TR+/TR- represents mosquitoes with infected or non-infected saliva, respectively. Kruskal-Wallis test: *, *P <* 0.05.

CHIKV, chikungunya virus; DENV, dengue virus; dpi, day post-infection; YFV, yellow fever virus; ZIKV, Zika virus.

When examining the viral load in abdomen according to different mosquito status (INF+/DIS+/TR+, INF+/DIS+/TR-, INF+/DIS-/TR-), no significant difference was detected between the three mosquito statuses except for YFV at 7 dpi (*P =* 0.016) as no mosquitoes were able to be infected, disseminate and transmit (Figure 2).

When considering all mosquitoes whatever the dpi and the virus, a significant correlation was found between abdomen and HT titers (Supplementary Figure 2a; rho = 0.67: *P <* 0.001), between HT and saliva titers (Supplementary Figure 2c; rho = 0.35: *P =* 0.002) but not between the abdomen and saliva titers (Supplementary Figure 2b; rho = 0.14: *P =* 0.22).

### Viral screening in field-collected mosquitoes

Using the BioMark microfluidic system, no targeted viruses were detected in 26 pools containing 47 mosquitoes (Supplementary Table 7).

## Discussion

CHIKV and ZIKV are the two viruses that have caused recent massive outbreaks in most continents including Latin America and the Caribbean. CHIKV first isolated in Tanzania in 1952 emerged outside Africa in 2004; the East-Central-South African genotype spread from Kenya to the Indian Ocean region, causing an epidemic on La Réunion Island. In October 2013, the first local cases of chikungunya were reported on Saint Martin Island in the Caribbean and affected most countries in the Americas. In 2014-2015, chikungunya affected Colombia with around 300,000 cases [11]. In 2023, CHIKV has caused outbreaks in Argentina, Paraguay, Bolivia, Brazil and Peru, with 125,867 confirmed cases and 291 deaths [11] where chikungunya mainly causes disability and economic burden given its chronic sequelae, weakening the health care systems [12]. In this study we showed that *Ae. aegypti* were highly susceptible to CHIKV with high IR, DR and TR suggesting that the two anatomical barriers to viral dissemination and transmission did not act as a brake to virus progression in the mosquito. Mosquito abdomen hosts fewer viral particles than HT, and saliva contain an average of 114 viral particles. Vector control interventions should be immediate once human cases are reported to contain viral dissemination as the mosquito can become infectious fewer than 7 days after an infectious blood meal [9]. Once well established, CHIKV can potentially initiate a sylvatic cycle as YFV did in the past. Two sylvatic Neotropical mosquito species, *Haemagogus leucocelaenus* and *Aedes terrens*, were highly competent for CHIKV, showing a vector competence similar to that of *Ae. aegypti* suggesting a high risk for CHIKV to establish a sylvatic transmission cycle in the Americas [13]. Furthermore, ZIKV first identified in 1947 in Uganda [14] emerged outside Africa and Asia in 2007 on Yap Island in Micronesia [15]; it was mainly transmitted by *Aedes hensilli*. After affecting islands in the Pacific region in 2013, Zika arrived in Brazil in 2013 [16] and spread to countries of Latin America including Colombia. In addition to more classical symptoms (fever, rash, arthralgia, conjunctivitis, pruritus, muscle pain, headache), Zika can cause severe manifestations, notably the Guillain-Barré syndrome and microcephaly of newborns; the circulating strains belonged to the Asian lineage [17]. The vectors were *Ae. aegypti* and *Ae. albopictus* were able to efficiently transmit ZIKV when co-infected with DENV. We showed that *Ae. aegypti* were susceptible to ZIKV with high IR and DR and lower TR compared to values with CHIKV meaning that the salivary glands play a stronger role as a barrier than the midgut. As for CHIKV, mosquito abdomen hosts less ZIKV particles than HT, but saliva contains a lower number of particles (an average of 69 viral particles). In Colombia, Zika seems causing shorter and intensive epidemics probably related to the high susceptibility of *Ae. aegypti* for ZIKV, which is confirmed by our study and goes against results obtained with Brazilian mosquitoes [18]; this confirms the virus-mosquito specific interactions governed by genotype-by-genotype (G-X-G) interactions, whereby the outcome of infection depends on the specific pairing of vector and pathogen genotypes [19]. Therefore, the effect of vector genes controlling competence depends on the pathogen genotype. However other factors can be incriminated such as people behavior who asked for more medical assistance because of association of ZIKV infection with congenital malformations and neurological abnormalities.

DENV and YFV are two other viruses introduced in the New World several centuries ago with DENV still causing massive outbreaks in Latin America and the Caribbean and YFV causing only sporadic human cases until its last emergence in Brazil. DENV was first identified in 1790 in Philadelphia, USA and later spread throughout the Americas, until the Pan American Health Organization set up a global eradication campaign against *Ae. aegypti* to control YFV in the 1950s and 1960s leading to elimination of the vector from most countries in the continent. After the relaxation of vector control measures in the early 1970s, the geographical distribution of *Ae. aegypti* is back to where it was before the eradication campaigns [20]. However, today, growing urbanization creates urban centers where there are more people and where the mosquito encounters ideal conditions for proliferation. This situation is aggravated by social, environmental and economic conditions that make vector control more challenging than it was in the past. The co-circulation of different DENV serotypes can increase the risk of severe DENV infection. The first major DENV hemorrhagic fever (DHF) epidemic in the Americas occurred in Cuba in 1981 and was caused by DENV-2. During the 2000-2006 period, 85,331 DHF cases were reported from Colombia and Venezuela [21]. In Colombia, DENV remains a major public health concern since 1989 [22]. We showed that *Ae. aegypti* were able to become infected, to disseminate and transmit DENV-1 with similar IR and DR suggesting that the midgut plays a weak role in preventing viral dissemination. TR values were much lower than IR and DR meaning that the salivary glands prevent viral transmission and saliva contains a low number of particles (an average of three viral particles). The other DENV serotypes can produce different results as *Ae. aegypti* shows differential susceptibilities to the four DENV serotypes. Moreover, YFV was isolated in West Africa in 1927 and introduced into the New World during the slave trade as was introduced the African mosquito *Ae. aegypti*. In the Americas, the disease caused devastating urban epidemics from the 18^th^ to the early 20^th^ century [23]. The development of two attenuated vaccines in the 1930s and a continental eradication program of *Ae. aegypti*, led to a clearance of urban YFV, which has not been reported in America since 1954 [24]. Today, in Latin America, human YFV infections are only acquired in the forest cycle [25]. YFV infections are usually mild but it can lead to a severe and often fatal disease. Despite the existence of an efficient vaccine, outbreaks have still been the cause of significant morbidity and mortality, especially in populations where vaccination coverage is low [26]. Colombia has suffered an important YFV outbreak in 2003, with 112 cases and 50 deaths reported [27]. We showed that *Ae. aegypti* were less susceptible to YFV with the lowest IR, DR and TR values compared to the three other viruses suggesting an efficient filtering at the midgut and salivary glands levels. However, mosquitoes excrete the highest number of viral particles in saliva (an average of 257 viral particles). Hence, only few mosquitoes were able to transmit YFV but viral loads in saliva are high, which likely increases the success of host infection.

To infect mosquito vectors, a minimum level of host viremia is necessary [28] and therefore, a minimum of viral loads in vectors is needed to trigger viral dissemination and transmission. We found no correlation between the viral load in mosquito abdomen and the success of viral dissemination and transmission unlike other combinations virus-mosquito [29] suggesting that other components may intervene in modulating viral transmission such as the microbiota [30]. Detection of viral infections in field-collected mosquitoes was also attempted and we used a mass screening method based on the BioMark™ Dynamic arrays system capable of detecting 37 different viral species in a single experiment. We screened 26 mosquito pools containing a total of 47 mosquitoes and failed to detect any circulating arboviruses, which was expected given the low number of mosquitoes examined.

These four human arboviruses are transmitted by the human-biting mosquito *Ae. aegypti* whose distribution has covered the tropical and subtropical regions (34). This mosquito is omnipresent in Colombia where DENV has been endemic since the 1970s with a co-circulation of the four serotypes since the 1980s [31], CHIKV and ZIKV hit the country in 2014 and 2015 respectively (35) and YFV causes sporadic cases since its introduction during the slave trade. In combination with a vaccine, efforts should be invested in prevention and control strategies with direct efforts toward the elimination of breeding sites and developing innovative environmental-friendly vector control strategies beside proposing new insecticide molecules. In conclusion, our study demonstrates that *Ae. aegypti* from Medellín was susceptible to the four most important arboviruses affecting humans with chikungunya being the most serious public health threat according to our vector competence data.

## Declarations of competing interest

The authors have no conflict of interest to declare.

## Appendix Supplementary materials

Image, application 1
