# Supplementary material for: Increased threat of urban arboviral diseases from Aedes aegypti mosquitoes in Colombia
Source: IJID Reg. 2024 Mar 22;11:100360. doi: 10.1016/j.ijregi.2024.100360 (PMC11002806; doi:10.1016/j.ijregi.2024.100360)
Supplement: Supplementary file 1 [file mmc1.docx]

**Table S1.** Details on collection sites in the northeastern part of Medellin city in Colombia. Mosquito eggs collected were pooled to constitute the Nor Oriental population.

| **Area** | **Commune Code** | **Commune** | **Neighborhood** | **Geographic Coordinates** | |
| --- | --- | --- | --- | --- | --- |
|  |  |  |  | **Latitude** | **Longitude** |
| 1 | 104 | Popular | Granizal | 837374.18 | 1187825.14 |
| 1 | 104 | Popular | Santo Domingo Savio No. 1 | 837836.88 | 1187812.17 |
| 2 | 203 | Santa Cruz | Pablo VI | 836648.16 | 1188945.97 |
| 2 | 203 | Santa Cruz | Pablo VI | 836653.28 | 1188964.26 |
| 2 | 203 | Santa Cruz | La Isla | 836873.54 | 1189028.2 |
| 2 | 209 | Santa Cruz | Moscú No. 1 | 836399.53 | 1187623.66 |
| 2 | 209 | Santa Cruz | Moscú No. 1 | 836456.95 | 1187792.98 |
| 2 | 209 | Santa Cruz | Moscú No. 1 | 836378.29 | 1187466.16 |
| 2 | 210 | Santa Cruz | Santa Cruz | 835931.37 | 1187756.05 |
| 2 | 210 | Santa Cruz | Santa Cruz | 835945.46 | 1187814.33 |
| 2 | 210 | Santa Cruz | Villa Niza | 836079.37 | 1188036.42 |
| 3 | 304 | Manrique | Santa Inés | 837185.16 | 1185345.62 |
| 3 | 304 | Manrique | Santa Inés | 837123.84 | 1185332.21 |
| 3 | 304 | Manrique | Las Granjas | 837291.94 | 1185998.07 |
| 3 | 304 | Manrique | Campo Valdés No. 2 | 836762.72 | 1185407.11 |
| 3 | 304 | Manrique | Campo Valdés No. 2 | 837039.84 | 1185650.34 |
| 4 | 402 | Aranjuez | San Isidro | 836083.67 | 1186949.33 |
| 4 | 402 | Aranjuez | San Isidro | 836016.6 | 1186952.87 |
| 4 | 402 | Aranjuez | San Isidro | 836023.29 | 1187091.94 |
| 4 | 406 | La Candelaria | El Chagualo | 835091.45 | 1184714.38 |
| 4 | 414 | Aranjuez | Campo Valdés No. 1 | 836088.5 | 1185415.68 |
| 4 | 414 | Aranjuez | Campo Valdés No. 1 | 836139.98 | 1185712.95 |
| 4 | 414 | Aranjuez | Brasilia | 836019.82 | 1185735.18 |
| 4 | 416 | Aranjuez | Jardín Botánico | 835411.08 | 1185208.33 |

**Table S2.** Nucleotide and amino-acid sequence differences between viral strains used in this study and strains circulating in Colombia

| Virus | Strain used | GenBank accesión # | Sequence used for comparison | % nucleotide differences | Number (%) of amino acid differences in envelope proteins |
| --- | --- | --- | --- | --- | --- |
| DENV-1 | French Guiana 2009 | MH279620 | R129 (2016) unpublished | 4.51% | 4 (0.81) |
| YFV | Bolivia 1999 | MF004382 | V-528A (1979) U23580 | 10.4%* | 15 (1.01) |
| CHIKV | La Reunión 2006 | AM258992 | Colombie 005-G6P5F7 unpublished | 6.3% | 28 (1.08)** |
| ZIKV | Martinique 2015 | KU647676 | UF-1 (2016) KX247646 | 0.18% | 1. (0) |

* E protein

** E1 and E2 glycoproteins

**Table S3.** Effect of virus and dpi on Log_10_ abdomen titer (linear regression model with interaction terms)

| Virus and Dpi | N | Mean (95% CI) abdomen titer (Log_10_) | p |
| --- | --- | --- | --- |
| CHIKV  7  14  21 | 24  24  22 | 4.86 (4.59-5.13)  4.89 (4.58-5.19)  3.65 (3.35-3.95) | **<0.001** |
| DENV  7  14  21 | 24  24  24 | 3.32 (2.99-3.64)  3.64 (3.36-3.93)  3.61 (3.25-3.96) | 0.29 |
| YFV  7  14  21 | 24  24  24 | 2.97 (2.51-3.43)  4.04 (3.68-4.39)  3.89 (3.54-4.23) | **0.001** |
| ZIKV  7  14  21 | 24  24  24 | 4.12 (3.82-4.41)  5.05 (4.75-5.34)  5.21 (4.91-5.51) | **<0.001** |

Dpi, day post-infection; N, number of mosquitoes tested; CI, confidence interval; in bold: significant p-values.

**Table S4.** Effect of virus and dpi on Log_10_ HT titer (linear regression model with interaction terms)

| Virus and Dpi | N | Mean (95% CI) HT titer (Log_10_) | p |
| --- | --- | --- | --- |
| CHIKV  7  14  21 | 22  18  18 | 5.15 (4.77-5.53)  5.18 (4.79-5.58)  4.97 (4.67-5.28) | 0.69 |
| DENV  7  14  21 | 11  16  12 | 2.39 (2.00-2.78)  3.09 (2.77-3.41)  3.14 (2.76-3.51) | **0.009** |
| YFV  7  14  21 | 2  4  8 | 2.83 (1.92-3.75)  3.22 (2.56-3.88)  4.32 (3.86-4.78) | **0.002** |
| ZIKV  7  14  21 | 8  18  17 | 2.76 (2.28-3.24)  4.43 (4.03-4.84)  5.23 (4.83-5.63) | **<0.001** |

Dpi, day post-infection; N, number of mosquitoes tested; CI, confidence interval; in bold: significant p-values.

**Table S5.** Infection, dissemination, and transmission rates of *Aedes aegypti* Nor Oriental exposed to blood meals containing CHIKV, DENV, YFV or ZIKV provided at a titer of 10^7^ FFU/mL. Mosquitoes were examined at day 7, 14 and 21 post-infection.

| Dpi | | CHIKV | DENV | YFV | ZIKV |
| --- | --- | --- | --- | --- | --- |
|  | 7 | 95.83 (24) | 66.66 (24) | 33.33 (24) | 79.16 (24) |
| IR | 14 | 75.0 (24) | 83.33 (24) | 54.16 (24) | 79.16 (24) |
|  | 21 | 86.36 (22) | 54.16 (24) | 58.33 (24) | 79.16 (24) |
|  | 7 | 95.65 (23) | 68.75 (16) | 25 (8) | 42.10 (19) |
| DR | 14 | 100 (18) | 80.0 (20) | 30.76 (13) | 94.73 (19) |
|  | 21 | 94.73 (19) | 92.30 (13) | 57.14 (14) | 89.47 (19) |
|  | 7 | 72.72 (22) | 36.36 (11) | 0 (2) | 12.5 (8) |
| TR | 14 | 77.77 (18) | 37.5 (16) | 25 (4) | 38.88 (18) |
|  | 21 | 66.66 (18) | 33.33 (12) | 25 (8) | 58.82 (17) |

IR, proportion of mosquitoes with infected abdomen (i.e. infected midgut); DR, proportion of mosquitoes with viral particles detected in head+thorax among mosquitoes with infected abdomen; TR, proportion of mosquitoes with viral particles in saliva among mosquitoes with infected head+thorax.

**Table S6.** Number of infectious viral particles (Log_10_) in body, head+thorax and saliva of *Aedes aegypti* Nor Oriental exposed to blood meals containing CHIKV, DENV, YFV or ZIKV provided at a titer of 10^7^ FFU/mL. Mosquitoes were examined at day 7, 14 and 21 post-infection.

| Dpi | | CHIKV | DENV | YFV | ZIKV |
| --- | --- | --- | --- | --- | --- |
|  | 7 | 5.26 (23) | 3.58 (16) | 3.61 (8) | 4.30 (19) |
| Body | 14 | 5.10 (18) | 3.96 (20) | 4.62 (13) | 5.52 (19) |
|  | 21 | 4.02 (19) | 3.87 (13) | 4.21 (14) | 5.57 (19) |
|  | 7 | 5.78 (22) | 3.23 (11) | 3.68 (2) | 4.04 (8) |
| Head+Thorax | 14 | 5.57 (18) | 3.29 (16) | 4.11 (4) | 5.27 (18) |
|  | 21 | 5.18 (18) | 3.33 (12) | 4.63 (8) | 5.87 (17) |
|  | 7 | 2.66 (16) | 0.70 (4) | - (0) | 0.90 (1) |
| Saliva | 14 | 2.32 (14) | 1.45 (6) | 0.30 (1) | 1.33 (7) |
|  | 21 | 2.06 (12) | 0.48 (4) | 2.41 (2) | 1.84 (10) |

**Table S7.** Pools of mosquitoes used for screening arboviruses using the BioMark Dynamic arrays system (Fluidigm Corporation) which targets 95 different genotypes/ serotypes of 37 viral species.

| Pool | Number of mosquitoes | Species | Details | Viral detection using the BioMark microfluidic system |
| --- | --- | --- | --- | --- |
| 1 | 1 | AA | 1 F | - |
| 2 | 1 | AA | 1 M | - |
| 3 | 1 | AA | 1 F | - |
| 4 | 8 | AA | 7 F + 1 M | - |
| 5 | 1 | AA | 1 F | - |
| 6 | 2 | AA | 2 F | - |
| 7 | 3 | AA | 2 F + 1 M | - |
| 8 | 4 | AA | 4 F | - |
| 9 | 1 | AA | 1 F | - |
| 10 | 2 | AA | 1 F | - |
|  |  | AL | 1 F | - |
| 11 | 1 | AA | 1 F | - |
| 12 | 2 | AA | 1 F + 1 M | - |
| 13 | 1 | AA | 1 F | - |
| 14 | 3 | AA | 2 F + 1 M | - |
| 15 | 3 | AA | 2 M + 1 F | - |
| 16 | 1 | AA | 1 F | - |
| 17 | 1 | AA | 1 F | - |
| 18 | 1 | AA | 1 F | - |
| 19 | 1 | AA | 1 F | - |
| 20 | 2 | AA | 2 F | - |
| 22 | 2 | AA | 2 F | - |
| 23 | 1 | AA | 1 M | - |
| 24 | 1 | AA | 1 M | - |
| 25 | 2 | AA | 1 M | - |
| 26 | 2 | AA | 1 M | - |
|  |  |  | 1 F | - |

AA, *Aedes aegypti*; AL, *Aedes albopictus*; F, female; M, male.

**Figure S1.** ROC curves to identify mosquitoes able to disseminate the virus according to abdomen_titer (a), and mosquitoes able to transmit the virus according to HT_titer (b).

**a**


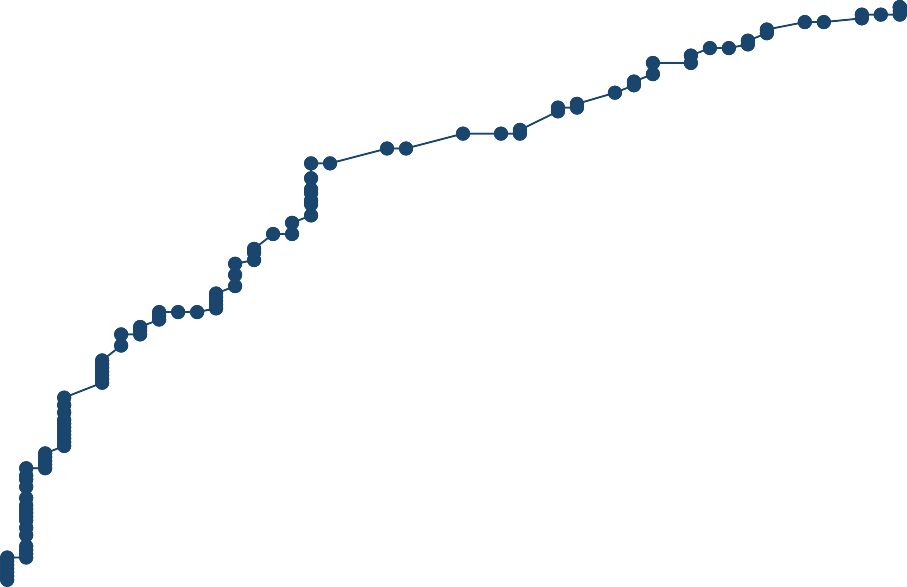

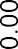

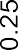

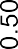

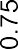

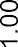

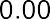

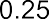

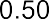

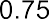

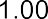

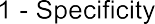

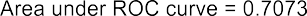


**b**


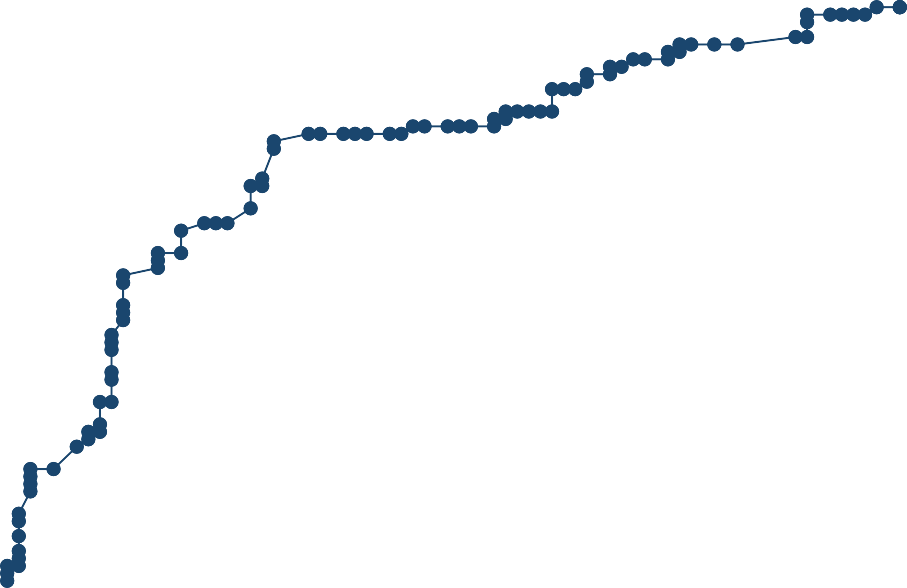

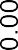

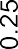

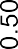

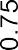

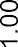

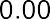

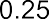

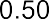

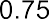

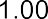

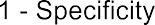

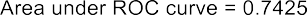


**Figure S2.** Correlation between abdomen_titer and HT_titer (a), abdomen_titer and saliva_titer (b), and between HT_titer and saliva_titer (c).

**a**

**8**


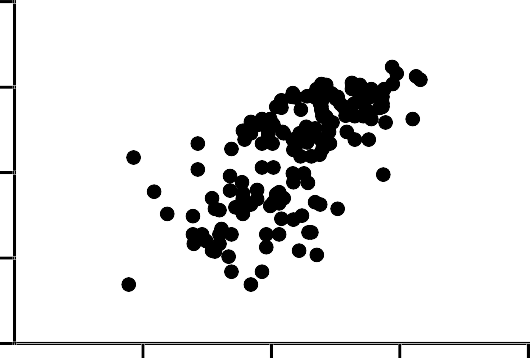


rho=0.67, p<0.001, N=154

**6**

Log_10__HT_titer

**4**

**2**

**0**

**0 2 4 6 8**

Log10_abdomen_titer

**b**

**4**


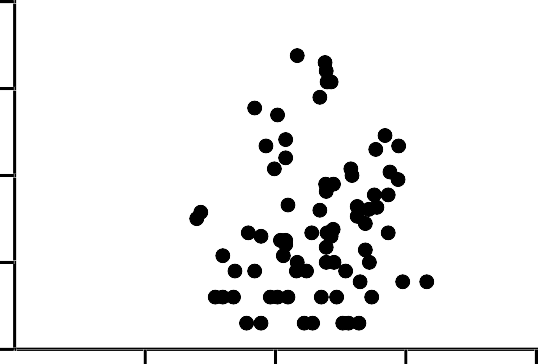


rho=0.14, p=0.22, N=77

**3**

Log_10__saliva_titer

**2**

**1**

**0**

**0 2 4 6 8**

Log10_abdomen_titer

**c**

**4**


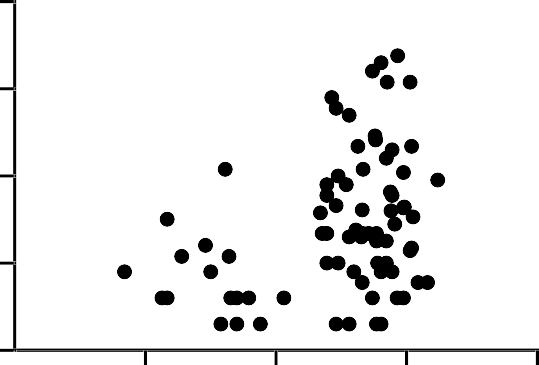


rho=0.35, p=0.002, N=77

**3**

Log_10__saliva_titer

**2**

**1**

**0**

**0 2 4 6 8**

Log10_HT_titer
